# Increased virulence of the oral microbiome in oral squamous cell carcinoma revealed by metatranscriptome analyses

**DOI:** 10.1038/s41368-018-0037-7

**Published:** 2018-11-12

**Authors:** Susan Yost, Philip Stashenko, Yoonhee Choi, Maria Kukuruzinska, Caroline A. Genco, Andrew Salama, Ellen O. Weinberg, Carolyn D. Kramer, Jorge Frias-Lopez

**Affiliations:** 1 Forsyth Institute, 245 First Street, Cambridge, MA 02142 USA; 2 Boston University Henry M. Goldman School of Dental Medicine, 100 East Newton Street, Boston, MA 02118 USA; 3 Department of Integrative Physiology and Pathobiology, Tufts University School of Medicine, 136 Harrison Avenue, Boston, MA 02111 USA; 4 Department of Oral Biology, College of Dentistry, University of Florida, 1395 Center Drive, Gainesville, FL 32610-0424 USA

## Abstract

Oral squamous cell carcinoma (OSCC) is the most prevalent and most commonly studied oral cancer. However, there is a void regarding the role that the oral microbiome may play in OSCC. Although the relationship between microbial community composition and OSCC has been thoroughly investigated, microbial profiles of the human microbiome in cancer are understudied. Here we performed a small pilot study of community-wide metatranscriptome analysis to profile mRNA expression in the entire oral microbiome in OSCC to reveal molecular functions associated with this disease. *Fusobacteria* showed a statistically significantly higher number of transcripts at tumour sites and tumour-adjacent sites of cancer patients compared to the healthy controls analysed. Regardless of the community composition, specific metabolic signatures were consistently found in disease. Activities such as iron ion transport, tryptophanase activity, peptidase activities and superoxide dismutase were over-represented in tumour and tumour-adjacent samples when compared to the healthy controls. The expression of putative virulence factors in the oral communities associated with OSCC showed that activities related to capsule biosynthesis, flagellum synthesis and assembly, chemotaxis, iron transport, haemolysins and adhesins were upregulated at tumour sites. Moreover, activities associated with protection against reactive nitrogen intermediates, chemotaxis, flagellar and capsule biosynthesis were also upregulated in non-tumour sites of cancer patients. Although they are preliminary, our results further suggest that *Fusobacteria* may be the leading phylogenetic group responsible for the increase in expression of virulence factors in the oral microbiome of OSCC patients.

## Oncology: bacteria may contribute to oral cancer development

A search of the oral microbiome has revealed a group of bacteria closely associated with the most common type of oral cancer, and whose activity may play a role in its development. Oral squamous cell carcinoma (OSCC) has been linked to tobacco and alcohol consumption, but there’s increasing interest in the role bacteria might play in its initiation and growth. To better understand the behaviour of the various microbes present in the mouths of healthy people and those with OSCC, Jorge Frias-Lopez at the University of Florida in Gainesville and his colleagues carried out a metatranscriptome analysis, which identifies the most actively expressed genes. This revealed significant differences in their microbial communities, with *Fusidobacteria* particularly enriched and hyperactive in patients with OSCC. The team also identified microbial activities that were consistently associated with cancer.

## Introduction

Oral squamous cell carcinoma (OSCC) is the most common malignancy of the head and neck, excluding non-melanoma skin cancer. In the United States alone, the American Cancer Society has estimated that there were 48 330 new cases of oral cavity and pharyngeal cancer in 2016, and of those, 31 910 were within the oral cavity itself.^1^

The two most commonly known aetiologic factors in oral cancers are tobacco and alcohol use. Human papillomavirus (HPV) has also been identified as a causal agent for oropharyngeal cancer in 4.4%–5.9% of cases.^2,3^ Although most studies assessing the infectious aetiology of cancer are focused on viruses, recently there has been increased interest in the possible role of the human bacterial microbiome in cancer.^4–8^ Chronic infections contribute to carcinogenesis, with approximately 18% of the global cancer burden being directly attributable to infectious agents.^7^ The association between chronic inflammation, oxidative stress and cancer is now well established.^9–11^ This association has recently received renewed interest with the recognition that microbial pathogens can contribute to the chronic inflammation observed in many cancers.^12^ In contrast, there is a void in knowledge regarding the role that microbiomes may play in carcinogenesis.^7^ Among them, the contribution of the oral microbiome is clearly understudied. In the case of OSCC, approximately 15% of oral cancer risk remains unexplained.^7,13^

Several studies have examined the associations between oral OSCC and colonisation with specific bacterial taxa/species.^14–17^ However, no robust and reproducible associations have emerged, and putative mechanisms of tumour promotion have yet to be defined. Furthermore, it is unclear whether shifts in the composition of the oral microbiome and chronic bacterial infection may promote cancer development, or if changes in the bacterial composition result from changes in the oral environment due to cancer. In contrast to composition assessments, metatranscriptomic analysis characterises community-wide gene expression profiles based on the set of transcripts being synthesised by the microbial community under diverse conditions. This approach allows for the assignment of activities to specific organisms in the transition from health to cancer, by unveiling the functional activities of these organisms in situ. Metatranscriptomics of the oral microbiome has been extremely informative in providing new insights into microbial functions and active communities in caries,^18,19^ periodontitis^20–22^ and during biofilm formation and after meal ingestion.^23^ These active communities were described based on the relative number of transcripts belonging to the different species in the community.

In the present pilot study, we used metatranscriptomic analysis to characterise bacterial functional activities in subjects with and without OSCC. OSCC sites were compared in a cross-sectional design to adjacent tumour-free sites of OSCC subjects and to matching sites from tumour-free controls.

## Results

Sequencing data were collected from 15 samples, including four tumour sites from OSCC subjects, four tumour-adjacent sites from OSCC subjects, four sites from healthy patients who matched the locations of the tumour sites and three buccal sites in healthy tumour-free subjects that matched the locations of tumour-adjacent samples. We used the R package RNASeqPower^24^ to estimate the target effect size needed to have significance with a false discovery rate (FDR) < 0.05 and power of 0.8, resulting in a minimum sample size of four individuals per group (Supplementary Table 1). The characteristics of the samples, as well as the number of total sequences and unique mapped reads are summarised in Supplementary Table 2.

### Active oral microbial communities associated with OSCC microenvironments are distinct from those of healthy controls

**Fig. 1:**
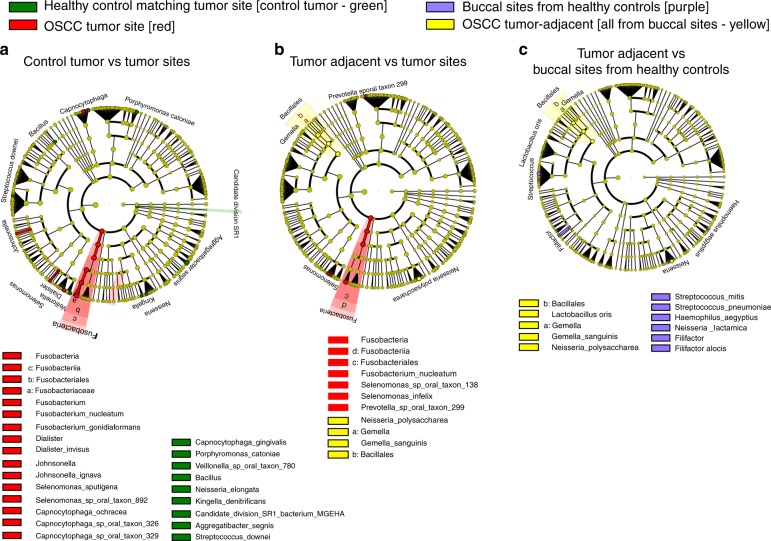
Statistical differences in the phylogenetic composition of active communities according to LEfSe. Cladograms report the taxa showing different abundance values for the transcripts (according to LEfSe). Metatranscriptome hit counts were obtained using Kraken against an oral microbiome database. Counts were then analysed using LEfSe to identify significant differences at the species level between the microbial communities compared. The alpha values were 0.05 for the Kruskal-Wallis (KW) sum-rank test and 0.01 for the Wilcoxon test. Only taxa with an LDA > 2 are represented in the cladograms. **a** Comparison of healthy control tumour-matched sites vs OSCC tumour sites. **b** Comparison of OSCC tumour-adjacent sites vs OSCC tumour sites. **c** Comparison of OSCC tumour-adjacent sites vs buccal sites from healthy control patients

We first compared the composition of the active communities based on the number of transcripts to identify the statistically significant differences between active groups. The most notable changes in the composition of active communities were observed in the comparisons between OSCC tumour sites and location-matched oral sites from healthy subjects (Fig. 1a). *Fusobacteria*, *Selenomonas* spp., *Capnocytophaga* spp. and members of the genera *Dialister* and *Johnsonella* were significantly more active in the tumour sites, while the genus *Bacillus* and the species *Porphyromonas catoniae*, *Kingella denitricans*, *Capnocytophaga gingivalis*, *Neisseria elongata*, bacterium MGEHA from the candidate division SR1, *Veillonella* sp. oral taxon 780, *Aggregatibacter segnis* and *Streptococcus downei* were more active in the healthy control sites (Fig. 1a).

Comparing the active microbiomes of tumour sites and tumour-adjacent sites in OSCC patients, *Fusobacteria*, *Selenomonas* spp. and *Prevotella* sp. oral taxon 299 were significantly more active in the tumour sites. In contrast, the order *Bacillales*, and the species *Gemella sanguinis* and *Neisseria polysaccharea* were more active in tumour-adjacent sites than in the tumour sites (Fig. 1b).

We also compared the metatranscriptomes from buccal sites in tumour-free subjects and tumour-adjacent sites from OSCC subjects to determine the impact of OSCC on oral sites distant from the tumour. As in the previous comparison, *Bacillales*, and the species *G. sanguinis* and *N. polysaccharea* were more active in tumour-adjacent sites from OSCC patients, indicating that those organisms are highly active at non-tumour locations in individuals with OSCC. In contrast, members of the genera *Filifactor* and *Streptococcus*, and the species *Neisseria lactamica* and *Haemophilus aegyptius* were more active in buccal sties from healthy controls.

Finally, we compared the location-matched control sites and buccal sites from OSCC patients, which represents the background differences expected between two oral sites of the same individual. Only two organisms showed elevated transcriptional activity that overlapped with the results of the other comparisons, including *Streptococcus pneumoniae*, which was more active in the buccal control sites, and *A. segnis*, which was more active in the location-matched control sites (Supplementary Fig. 1). Accordingly, the differences observed for these two organisms in the remaining comparisons were not analysed further.

### Metatranscriptomic analysis of the oral microbiome reveals functional activities associated with OSCC

**Fig. 2:**
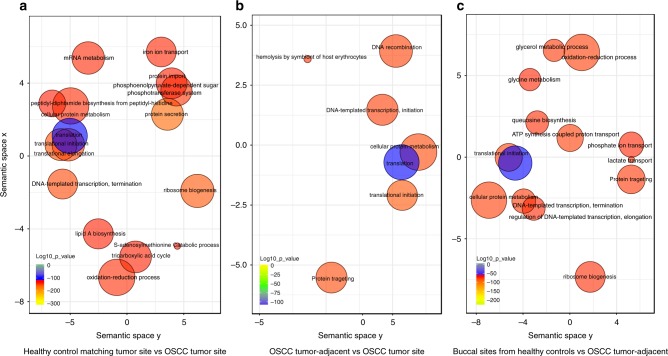
GO enrichment analysis of the metatranscriptome profiles of the oral microbiome associated with cancer status; over-represented biological processes. Enriched terms obtained using GOseq were summarised and visualised as a scatter plot using REVIGO. **a** Summarised GO terms in healthy control tumour-matched sites vs OSCC tumour sites. **b** Summarised GO terms in OSCC tumour-adjacent sites vs OSCC tumour sites. **c** Summarised GO terms in OSCC tumour-adjacent sites vs buccal sites from healthy control patients. Bubble size indicates the frequency of the GO term in the underlying GO database. Colour indicates the log10 *P*-value (red higher, green lower) showing the significance of GO enrichment for a particular GO term. The *X* and *Y* axes represent semantic spaces that have no intrinsic meaning. REVIGO uses multi-dimensional scaling to reduce the dimensionality of a matrix of the GO terms’ pairwise semantic similarities. The results with semantically similar GO terms remain close together in the plot. Semantic similarity between two GO terms is based on the shared proteins contained in those terms

We identified the differentially expressed genes using two methods: NOISeq and GFOLD. Only genes that were identified as differentially expressed using both methods were subjected to further analysis. We characterised the global behaviour of the community based on the Gene Ontology (GO) terms that were significantly enriched. Comparing tumour sites with location-matched non-tumour sites, GO biological process enrichment revealed several over-represented activities, including iron ion transport (Fig. 2a). When tumour sites were compared to tumour-adjacent sites in OSCC patients, over-represented terms were related to haemolysin activity on host erythrocytes (Fig. 2b). Moreover, tumour-adjacent sites from tumour patients had over-represented activities associated with glycine metabolism and phosphate and lactate transport when compared with buccal healthy control sites (Fig. 2c). The only significant under-represented activity at the level of biological processes between tumour sites and controls was nucleotide excision repair (Supplementary Fig. 2). Most importantly, despite differences in phylogenetic profiles representing the active communities shown above, there were no significant differences in GO enrichment terms between healthy control-matched tumour sites and the buccal sites from healthy control samples (Fig. 2).

Supplementary Figs. 3 and 5 summarise the results of the enrichment of GO term-related molecular functions. Activities including iron ion binding, tryptophanase activity, glutamate dehydrogenase (GDH), starch synthase activity and superoxide dismutase (SOD) were over-represented at tumour sites and tumour-adjacent samples when compared to the healthy tumour-free controls (Supplementary Figs. 3 and 5, Supplementary Table 3).

Specific molecular functions associated with iron binding (iron, ferric ion and haemin), as well as nitrous-oxide reductase, metalloexopeptidase and lactoylglutathione lyase activity, were higher in tumour sites compared to control location-matched sites and tumour-adjacent sites from OSCC patients (Supplementary Figs. 3 and 4, Supplementary Table 4). Interestingly, when comparing tumour-adjacent sites from OSCC patients with buccal sites from non-tumour controls, we observed activities at tumour-adjacent sites that were also associated with the microbiome at tumour sites, suggesting widespread dysbiosis in the oral cavity of OSCC patients (Supplementary Fig. 5, Supplementary Table 4).

**Fig. 3:**
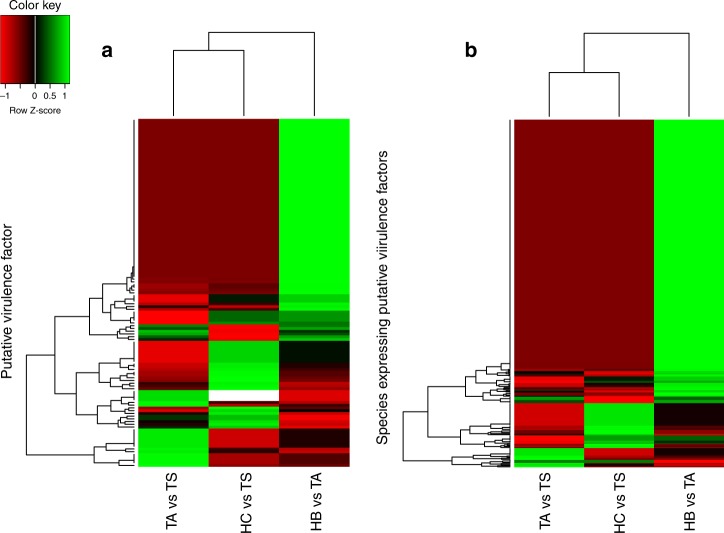
Heatmaps of putative virulence factor analysis. Upregulated putative virulence factors were used for cluster analysis. **a** Cluster analysis of upregulated virulence factor expression profiles based on the levels of expression of specific virulence genes. **b** Cluster analysis of upregulated virulence factor expression profiles based on the levels of expression observed for specific species of bacteria in the biofilm. TS, OSCC tumour site; HC, healthy control tumour-matched site; TA, OSCC tumour-adjacent site; HB, buccal sites from healthy controls

We also analysed the expression of putative virulence factors in the oral communities associated with different OSCC statuses (Supplementary Table 5). Comparing tumour sites either with location-matched sites from healthy patients or tumour-adjacent sites from OSCC patients, the fraction of differentially expressed putative virulence factors was between 0.35% and 0.41% of the total number of differentially expressed genes (Supplementary Table 5). Interestingly, buccal non-tumour sites from OSCC patients showed a higher proportion of upregulated virulence factors when compared to healthy buccal controls. Only 1.91% of all differentially expressed genes were identified as putative virulence factors in this case (Supplementary Table 5). Clustering the profiles of expression of the differentially expressed virulence factors in the different comparisons, we observed that the patterns of the buccal healthy control sites vs. tumour-adjacent sites from OSCC patients were distinct from the patterns observed when tumour sites were compared (Fig. 3a). Concordantly, when we clustered the differentially expressed putative virulence factors based on their phylogenetic origin, we obtained a similar profile (Fig. 3b). Taken together, these findings indicated that the taxa responsible for most of the putative virulence factor expression at tumour sites was *Fusobacteria*.

Comparing tumour-adjacent sites with healthy buccal controls, we observed activities associated with protection against reactive nitrogen intermediates (AhpC), chemotaxis (cheA and cheY), urease activity (ureG) and serine-type protease activity (mycP2) (Supplementary Table 5). At tumour sites, regardless of the control used for comparison, we also observed the upregulation of virulence factors associated with chemotaxis (cheR and cheV), iron uptake activities (ferrous iron transporters, iron transport systems and yersiniabactin), protease activity (zinc metalloproteases, ATP-dependent Clp protease, serine protease and immunoglobulin protease), type II, III and IV secretion system proteins, oligopeptide ABC transporters, flagellum synthesis and assembly (FlgG, FliL and flagellar MS-ring protein), and fibronectin-fibrinogen-binding proteins (Supplementary Table 5).

**Fig. 4:**
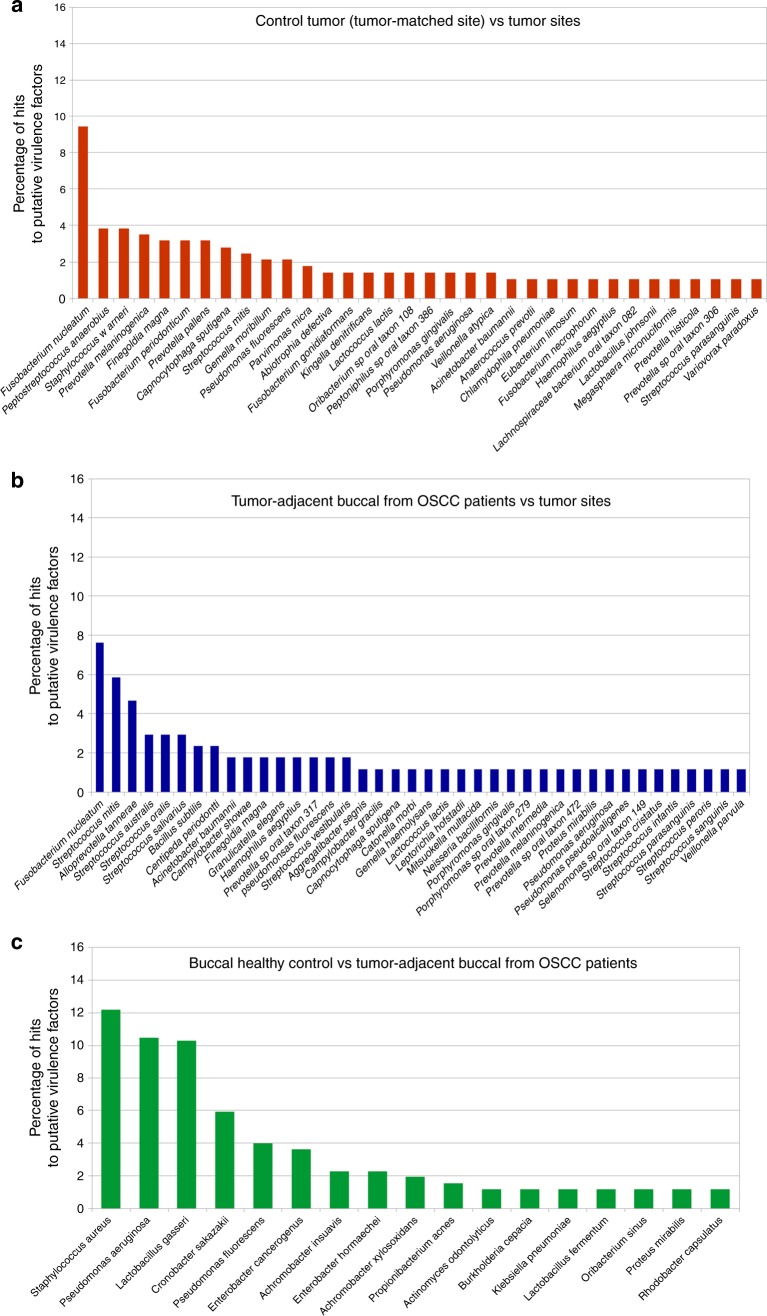
Phylogenetic origin of upregulated putative virulence factors. Relative abundance of hits from a specific bacterium of the total differentially expressed virulence factors in each of the comparisons. **a** Comparison of healthy control tumour-matched sites vs OSCC tumour sites. **b** Comparison of OSCC tumour-adjacent sites vs OSCC tumour sites. **c** Comparison of OSCC tumour-adjacent sites vs buccal sites from healthy control patients

Cluster analysis of the phylogenetic origins of the putative virulence factors indicated that the composition of community members expressing these factors was different in tumour and healthy sites. Thus, we found that *Fusobacterium nucleatum* was the most active bacterium expressing putative virulence factors in the tumour sites (Fig. 4a, b). In contrast, *Staphylococcus aureus* was the most active in putative virulence factor expression at buccal sites in OSCC patients compared to healthy buccal sites (Fig. 4c). Upregulated putative virulence factors from *F. nucleatum* represented 9.5% of the total number of hits in the tumour sites compared to location-matched sites in controls (Fig. 4a) and 7.6% of hits in tumour sites compared to buccal sites from OSCC patients (Fig. 4b)*. S. aureus* represented 12% of the total in the tumour-adjacent samples from OSCC patients when compared to healthy buccal-matched sites (Fig. 4c). Another *Fusobacteria*, *F. periodonticum* was also responsible for a sizable fraction of the upregulated putative virulence factors at tumour sites (Fig. 4a).

### Role of *Fusobacteria* in the activities of the oral microbiome associated with cancer status

**Fig. 5:**
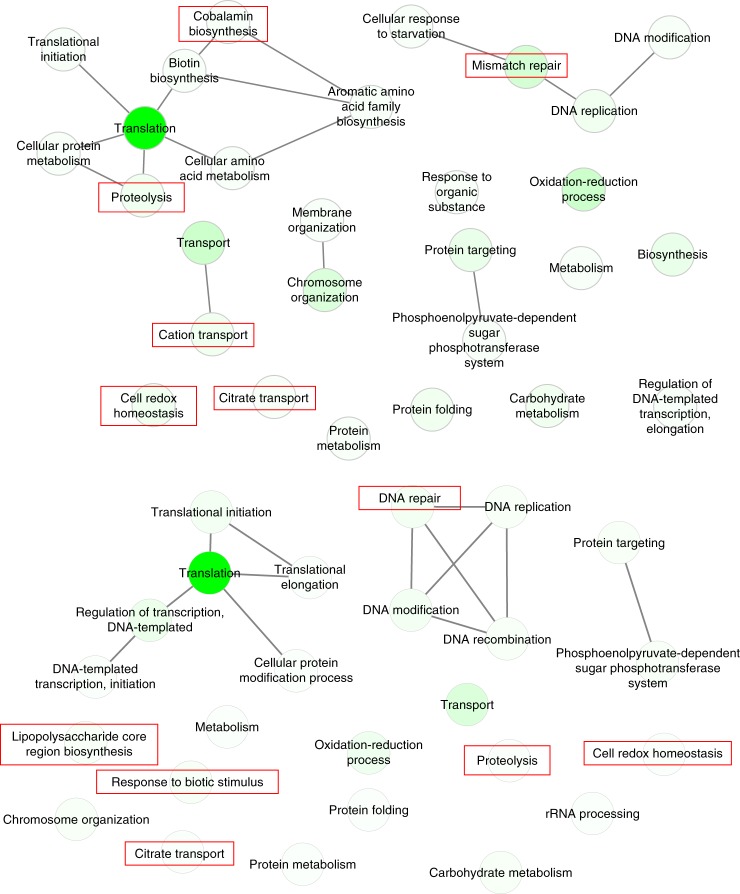
GO enrichment analysis of the metatranscriptome profiles of *Fusobacteria*; over-represented biological processes. Enriched terms obtained using GOseq were summarised and visualised as a network using REVIGO. **a** Summarised GO terms in healthy control tumour-matched sites vs OSCC tumour sites. **b** OSCC tumour-adjacent sites vs OSCC tumour sites. In red are activities that are also over-represented in the oral microbiome in periodontal disease^20,21^

Given that *Fusobacteria* were the best biomarkers for tumour sites based on expression analysis (Fig. 1a, b) and that *F. nucleatum* showed the highest upregulation of putative virulence factors (Fig. 4a, b), we investigated the metabolic activities that were associated with this taxonomic group. At tumour sites, when compared to either location-matched control sites or adjacent buccal sites in OSCC patients, proteolysis, DNA mismatch repair, carbohydrate metabolism, cell redox homeostasis and citrate transport were all over-represented (Fig. 5).

## Discussion

In the present study, we performed a pilot metatranscriptomic analysis of the oral microbiome associated with human OSCC sites. Given the limited number of patients, we focused our study on non-smoking and HPV-negative OSCC samples, thus reducing, to some extent, the variability due to other high-risk factors for OSCC. The current results represent a first glimpse at the metabolic changes that the oral microbiome undergoes during environmental conditions associated with OSCC. Nevertheless, clear changes in microbial metabolic activities were apparent in OSCC, regardless of the community composition. These metabolic activities included iron acquisition, response to oxidative stress and peptidase activity.

The microbiome compositions of different oral sites are quite specific.^25–27^ Although we analysed different oral sites in the present study, the controls used in this analysis matched the cancerous sites being compared, whereby each patient had its own control. Moreover, we did not observe significant location-associated differences in terms of the active communities or GO term enrichment analyses, as described in detail above.

Iron is an essential element in a significant number of physiological processes, including DNA synthesis and respiratory and oxidative metabolism. These metabolic activities are associated with cell proliferation and a more anaerobic metabolism, which may contribute to both tumour initiation and progression. Iron has long been implicated as playing a significant role in carcinogenesis and cancer progression.^28–30^ In the host, pathways of iron acquisition, efflux, storage and regulation have all been reported to be altered in cancer by other authors,^30^ and competition with a more virulent oral microbiome for this scarce resource may contribute to the deregulation of iron pathways in the oral epithelium. Moreover, iron represents a critical factor that determines the generation of reactive oxygen species (ROS), thereby influencing the outcome of ROS-induced effects.^31^

We also observed an increase in the activity of SOD in the microbiomes of both tumour sites and adjacent buccal sites from cancer patients. This indicated the presence of O_2_^.−^ at these sites, which is converted to H_2_O_2_ by SOD. In the presence of Fe^2+^, this ion can react with H_2_O_2_ leading to the production of extremely reactive intermediates. These reactive intermediates can contribute to the initiation and/or progression of cancer by oxidative DNA damage and to cancer cell proliferation through the modulation of cell cycle proteins such as p53 or by suppressing cell death.^32^ These findings are consistent with well-established associations between chronic inflammation, oxidative stress and cancer.^9,11^

The generation of microenvironments with high levels of ROS, such as those observed in our samples, could be a contributing factor to potential chronic DNA damage that could lead to OSCC in patients presenting no apparent risk factors.

The aryl hydrocarbon receptor (AHR) is a ligand-activated transcription factor best known for mediating the toxicity and tumour-promoting properties of dioxin. Nonetheless, many other compounds can act as AHR agonists and promote carcinogenesis.^33^ One specific activity of the microbiome that was found to be over-represented at tumour and tumour-adjacent buccal sites in OSCC patients was tryptophanase, which catalyses the degradation of l-tryptophan to indole, pyruvate and ammonium, all of which may be metabolised into agonists for AHR and potentially contribute to carcinogenesis. Significantly, a recent report found that the putative oral pathogen, namely, *P. gingivalis*, could produce AHR agonist activity.^34^

GDH was also over-represented at tumour and tumour-adjacent buccal sites in OSCC patients. This enzyme may play a pivotal role in glutamine metabolism in the microbiome and in the host. GDH is elevated in tumours and contributes to redox homeostasis in the cell.^35,36^ We observed a similar behaviour in the oral microbiome, possibly in response to the ROS in the environment. We also found an increase in lactoylglutathione lyase activity at tumour sites. This enzyme is responsible for the detoxification of methylglyoxal using glutathione as a substrate and producing (R)-*S*-lactoylglutathione as the final reaction product. In cancer cells, metabolic reprogramming towards aerobic glycolysis increases methylglyoxal levels. Methylglyoxal leads to the chemical modification of proteins, lipids and nucleotides that may induce cellular dysfunction and mutagenicity.^37^ Bacteria use lactoylglutathione lyase activity as a defence mechanism against the effects of methylglyoxal in the environment.^38,39^

Another activity from the oral microbiome that could be involved in carcinogenesis is the reduction of nitric-oxide (NO) by nitrite reductase, which we observed in buccal sites from OSCC patients when compared to buccal sites in controls. It has been proposed that low concentrations of NO can be pro-angiogenic and induce tumour growth, whereas high NO levels may have the opposite effect.^40,41^ As in the case of ROS discussed above, the sustained generation of NO could contribute to DNA damage that may be involved in those OSCC cases with no apparent risk factors.

As noted above, while the relationship between bacterial profiles and OSCC has been thoroughly studied, no clear association between microbial community composition and cancer status has emerged.^42^ A large number of different organisms have been identified as more abundant in or around OSCC samples, and among them are species of *Fusobacteria*,^15,17,43^ *Porphyromonas*,^43,44^ *Streptococcus anginosus*^45,46^ and several other *Streptococci*,^16,17^ *Prevotella*,^15^ *Peptostreptococcus*^15,47^ and *Bacteroidetes*.^17^ From the transcriptional perspective, however, we found that only *Fusobacteria* appeared to be metabolically hyperactive in the oral community of OSCC patients, in agreement with some previous studies and echoing findings in colorectal cancer.^48–50^ This taxa was highly active at OSCC sites, with over-represented metabolic activities that included proteolysis, iron ion transport and cobalamin biosynthesis, all of which are activities that we previously detected in dysbiotic oral biofilms in periodontitis.^21,51^ *Fusobacteria* furthermore showed a significant increase in overall virulence factor expression at those sites. In a recent study of 4-NQO-induced oral cancer, it was reported that both *P. gingivalis* and *F. nucleatum* were delivered in large quantities to the oral cavity and could promote tumour progression in mice, although persistent colonisation was not documented for either organism.^52^ Chronic *P. gingivalis* infection has been associated with orodigestive cancer,^53^ an increase in oral cancer invasion,^54^ epithelial to mesenchymal transitions^55^ and the production of oral cancer stem cells.^56^ However, in the present study, *P. gingivalis* appeared not to be hyperactive in OSCC, although a larger number of samples could reveal its potential importance.

Finally, an important aspect of the potential role of the microbiome in carcinogenesis is the expression of virulence factors by biofilms in the tumour microenvironment. Our findings showed a striking increase in the general expression of virulence genes in the tumour-associated microbiomes, both at tumour sites and at tumour-adjacent buccal sites, notably, by *Fusobacteria*. Virulence factors of *F. nucleatum* in conjunction with disruptions in epithelial signalling and the promotion of inflammation have been suggested as important elements for creating the ideal microenvironment for the progression of colorectal cancer.^57^ In addition, an enrichment in virulence-associated bacterial genes in the tumour microenvironment of colorectal cancer has been reported.^58^

Metatranscriptomics is emerging as a powerful approach for the functional characterisation of complex microbial communities. Here we report findings from a pilot study of the metatranscriptome of the oral microbiome associated with human OSCC, in which we have identified microbial activities of the biofilm in disease. Although our results show an association of microbial activities with a potential role(s) in cancer, causality cannot be implied by this study, especially given the limited number of samples analysed. Nonetheless, the data presented herein illustrate that certain overexpressed metabolic signatures are consistently related to cancer status. Among them, we observed an increase in the response to oxidative stress, iron acquisition and peptidase activities, as well as an increase in the overall expression of virulence factors. We also found that the metabolic activities of the microbiome in non-tumour buccal sites from cancer patients were also impacted and differed from the baseline activities of healthy tumour-free controls, suggesting oral microbial dysbiosis in OSCC. Echoing other studies on the role of the microbiome and cancer, *Fusobacteria* appeared to be a key player in the contribution of the microbiome to activities associated with OSCC. This work represents a first attempt to describe the oral metatranscriptome of the oral microbiome in OSCC. The analysis of larger cohorts should provide a better characterisation of the metabolic activities linked to OSCC, providing researchers and clinicians with potential targets for therapeutic intervention to improve patient outcomes.

## Materials and methods

### Ethics statement

Verbal informed consent was obtained from all participants in this study. Recruitment of study participants was performed according to the protocol approved by the Boston University Medical Campus Institutional Review Board H-31936 (Approval Date, 27 Aug 2014). All subjects provided verbal informed consent prior to participation.

### Study design, subject population and sample collection

We conducted a cross-sectional comparison of gene expression in subjects with and without OSCC. All study subjects, patients and controls were males, 40–64 years of age, who had >15 natural teeth and were in good general health. The patients with OSCC were eligible for this study if they had newly diagnosed non-metastatic squamous cell carcinoma of the oral cavity. All patients were HPV-negative. Healthy individuals were matched for age and sex with the OSCC patients and all were non-smokers. Subjects with OSCC were ineligible if they previously received any treatment, including surgery, radiotherapy and/or chemotherapy, if they were pregnant or nursing, received antibiotics or periodontal therapy in the previous 3 months, if they had any systemic illnesses, including diabetes, or if they had any immunocompromising conditions.

Oral swab samples were collected from four distinct sites. They were separately obtained from (i) the OSCC tumour site, (ii) a healthy control site from a healthy patient matching the tumour site, (iii) a healthy buccal site from a tumour-free healthy individual and (iv) an OSCC tumour-adjacent site (all from buccal sites) from a cancer patient. Each sample was placed in an individual tube containing 0.5 mL of RNAlater (Life Technologies, Grand Island, NY, USA) and stored frozen at −80 °C.

### Target effect size calculation per sample size

We used the R package, RNASeqPower,^24^ to estimate the target effect size needed to have significance with a FDR < 0.05 and power of 0.8, resulting in a minimum sample size of four individuals per group. RNASeqPower provides a theoretical estimate of power over a range of variables, given the within-group variances of the samples, which are intrinsic to the experiment^59^ and are independent of the type of transcriptome analysis performed, depending only on the genome coverage and the coefficient of variation (CV).^24^ We first estimated the average coverage using the SAMtools ‘mpileup’ command from the SAMtools package.^60^ For metatranscriptome analysis, the target effect size considered significant for our sample size was 2.75, except for the analysis of buccal healthy controls which, given that we had three patients, the cut-off was a 3.25-fold change to be considered significant (Supplementary Table 1).

### Microbiome community RNA extraction

RNAlater was gently removed from the tubes containing the swabs, and mirVana kit lysis/binding buffer (600 μL) and 0.1-mm zirconia-silica beads (BioSpec Products, Bartlesville, Okla) (300 μL) were added to the samples. Samples were bead-beaten for 1 min at maximum speed. Tips were removed after the bead-beating step and processed following the manufacturer’s instructions, except that 0.1% hydroxyquinoline was added to the phenol:chloroform solution to facilitate the distinction between the two phases. For metatranscriptome analysis, MICROB*Enrich* (Life Technologies) was used to remove eukaryotic RNA, and MICROB*Express* (Life Technologies) was used to remove prokaryotic rRNA, following the manufacturer’s instructions.

### RNA amplification and illumina sequencing

For microbiome metatranscriptomic analysis, RNA amplification was performed on total enriched bacterial RNA using the MessageAmp^TM^ II-Bacteria RNA amplification kit (Life Technologies) following the manufacturer’s instructions. Sequencing was conducted at the Forsyth Institute Sequencing Core. Illumina adapter-specific primers were used to amplify and selectively enrich for the cDNA generated from enriched mRNA. The TruSeq Stranded mRNA kit was used to generate libraries from amplified DNA. Samples were run using the NextSeq 500 using the 2 × 75 bp 150 cycle v2 reagent kit (Illumina). The samples were pooled in batches of up to 12 samples per run. The only variation in the original Illumina protocol was that the samples began the protocol following purification and fragmentation of the mRNA by adding approximately 400 ng in 5–13 μL of the Fragment Primer Finish Mix.

### Short reads sequence alignment analysis

Low-quality sequences were removed from the query files. The FASTQ Clipper and FASTQ Quality Filter programmes from the FASTX-toolkit (http://hannonlab.cshl.edu/fastx toolkit/) were used to save long sequences with a *Q* > 33 in >80% of the sequence. Cleaned files were then aligned against a customised bacterial/archaeal database, containing 549 genomes from 349 oral species, using Bowtie 2, with parameters -q --local -N 1 -L 20 -D 30 -t -R 3 -i S,1,0.25, as described in Duran-Pinedo et al.^20^ We generated a.gff file to map hits to different regions of the genomes in our database. Read counts from the SAM files were obtained using bedtools multicov from bedtools.^61^

### Phylogenetic analysis of the active communities based on their metatranscriptomes

Counts from the mRNA libraries were used to determine their phylogenetic composition for bacteria and archaea. Phylogenetic profiles of the metatranscriptomes were obtained using Kraken.^62^ We generated a custom Kraken library with the oral microbiome genomes indicated in the above section with a filtering threshold of 0.05.^63^ Phylogenetic profiles were used to identify significant differences between active communities under the different conditions studied by performing linear discriminant analysis effect size (LEfSe), as proposed by Segata et al.,^63^ with an alpha value for the Wilcoxon test of 0.01. Significant *P*-values associated with microbial clades and functions identified by LEfSe were corrected for multiple hypothesis testing using the Benjamini and Hochberg FDR correction^64^ using the p.adjust function in R with a cut-off FDR < 0.05.

### Differential gene expression analysis

To identify differentially expressed genes from the RNA libraries, we applied non-parametric tests to the normalised counts using the NOISeqBIO function of the R package. NOISeq conditions were as follows: *k* = 0.5, lc = 1, cv.cut-off = 50 (genes with a CV > 0.5 were discarded for analysis), replicates = “biological” and tmm normalisation (tmm option) with length correction. We also removed batch effects and used the threshold value for significance suggested by the authors of *q* = 0.95, which for the function NOISeqBio is equivalent to an FDR cut-off of 0.05.^65^ We used a cut-off fold change of 2.75 when four samples per group were compared and 3.25 when one of the two groups had three samples. The justification for the use of these values appears in the above section ‘Target effect size calculation per sample size’. Rarefaction results obtained using the NOISeq package for all three comparisons are shown in Supplementary Fig. 6.

We also performed differential expression analysis using GFOLD, which takes a Bayesian approach in which fold change is derived from the posterior distribution of the raw fold change.^66^ Only genes that were identified as differentially expressed in both NOISeqBio and GFOLD were used for further analysis. The number of differentially expressed genes used for further analysis was 35 404 genes for the control tumour vs. tumour site comparison, 41 421 genes for the control tumour-adjacent site vs. buccal sites from healthy controls comparison and 405 411 for the tumour-adjacent vs. tumour site comparison.

### GO enrichment analysis

To evaluate the functional activities that were differentially represented in healthy controls, buccal sites from cancer patients and tumour sites, we mapped the differentially expressed genes to known biological ontologies based on the GO project (http://www.geneontology.org/). GO terms to which the different open reading frames belonged were obtained from the PATRIC database (http://patricbrc.org/portal/portal/patric/Home). GO terms not present in the PATRIC database and whose annotation was obtained from the HOMD database or the J. Craig Venter Institute were acquired using the programme blast2GO under the default settings.^67^

Enrichment analysis of these data sets was performed using the R package GOseq, which accounts for biases due to over detection of long and highly expressed transcripts.^68^ Gene sets with ≤10 genes were excluded from analysis. We used the REVIGO web page^69^ to summarise and remove redundant GO terms from the results. Only GO terms with FDR < 0.05 were used. REVIGO plots were obtained for two categories (biological process and molecular function). In the case of specific organisms, we mapped upregulated genes to GO terms and ranked them before summarising the results using REVIGO. The plots were visualised either using the R script obtained from REVIGO or the network ‘.xmmgl’ file that can be opened and modified in Cytoscape 3.^70^

### Quantification of transcribed putative virulence factors

To identify putative virulence factors, we used the Virulence Factors of Pathogenic Bacteria Database (VFDB; http://www.mgc.ac.cn/VFs/). A similar approach, but with less stringent conditions, has been used by other authors to identify putative virulence factors in genomic islands.^71^ The VFDB contains 1 205 virulence factors and 5 955 virulence factor-related genes from 75 pathogenic bacterial genera.^72^ We performed a BLAST similarity search of encoded proteins from the genomes in our database against the VFDB, with an *e*-value cut-off of 10^−25^ and an identity score > 99% to exclude distant homologues.

We obtained a heatmap representation of the upregulated virulence factors and species that transcribed them across samples, using the R packages ‘vegan’ and ‘gplots’. Counts were normalised using the frequency transformation of the function ‘decostand’.^73^ Using the ‘heatmap.2’ function in R, we clustered samples and represented their heatmaps based on their expression profiles. The clustering function for ‘heatmap.2’ was ‘hclust’ selecting ‘complete’ (complete-linkage) as the clustering method.

## Electronic supplementary material

Bioinformatic analysis

Supplementary Figure 1

Supplementary Figure 2

Supplementary Figure 3

Supplementary Figure 4

Supplementary Figure 5

Supplementary Figure 6

Supplementary Table 1

Supplementary Table 2

Supplementary Table 3

Supplementary Table 4

Supplementary Table 5

## Data Availability

The data sets used in these analyses were deposited at the Human Oral Microbiome Database (HOMD) under the submission number 20161121 (ftp://www.homd.org/publication_data/20161121/).
