# Supplementary material for: Increased virulence of the oral microbiome in oral squamous cell carcinoma revealed by metatranscriptome analyses
Source: Int J Oral Sci. 2018 Nov 12;10(4):32. doi: 10.1038/s41368-018-0037-7 (PMC6232154; doi:10.1038/s41368-018-0037-7)
Supplement: Supplementary file 1 — Bioinformatic analysis [file 41368_2018_37_MOESM1_ESM.pdf]

```
########################################################################################
######################### BIOINFORMATIC ANALYSIS #######################################
########################################################################################

########################################################################################
####################### Removal of low-quality sequences ##################################
########################################################################################

$ fastx_clipper -i Sample_1_R1.fastq -o Sample_1_R1_clipped.fastq -l 50  -v -a
AAAAAAAAAAAAAAAAAAAAAAAAAAAAAAAAAAAA

$ fastq_quality_filter -i Sample_1_R1_clipped.fastq -o Sample_1_R1_clean.fastq -q 33 -p 80 -v

####### Combine the clean pair-end sequences ######

$ python fastqcombinepairedend.py  Sample_1_R1_clean.fastq Sample_1_R2_clean.fastq None

########################################################################################
##############################  bowtie2 aligment #########################################
########################################################################################

$ bowtie2 --local -N 1 -L 20 -D 30 -t -R 3 -i S,1,0.25 --no-unal -p40 –no-discordant -x
./oral_microbiome_genomes -1 Sample_1_R1_clean_trimmed_clipped_stillpaired.fastq -2
Sample_1_R1_clean_trimmed_clipped_stillpaired.fastq -S Sample_1.sam

$ samtools view -@20 -bS Sample_1.sam > Sample_1.bam

$ samtools sort -@35 Sample_1.bam Sample_1Sorted

$ samtools index Sample_1Sorted.bam

$ bedtools multicov -bams Sample_1Sorted.bam .... Sample_NSorted.bam -bed oral_microbiome_CDS.gff >
gene_counts.gff ##### Generates the counts table

$ sed 's/^.*locus_tag=//' gene_counts.gff > gene_counts.tab

########################################################################################
##################### Differential expression analysis with GFOLD #############################
########################################################################################


$  awk -v OFS='\t' '{ print $1,$2,$3,$4,$5 > "Condition1.read_cnt"; print $1,$6,$7,$8,$9 > "Condition2_cnt" }'
gene_counts.tab # Generates *_cnt files from counts table.

$  gfold diff -s1 Condition1.1,Condition1.2 … -s2 Condition2.1, Condition2.2 ... -suf .read_cnt -o
sample1VSsample2.diff


########################################################################################
################### NOISeqBio in R Exploratory analysis ####################################
########################################################################################

> library(NOISeq)
```

```
> mycounts<-read.table('gene_counts.tab', header=TRUE, row.names=1)

> mycounts=mycounts[which(rowSums(mycounts) > 0),] # Remove 0S

> mylength<-read.table('gene_length_NOISeq.txt', header=FALSE) ######## In case you use RPKM or TMM
length correction

> myfactors = data.frame(condition =
c("Condition1","Condition1","Condition1",..."Condition2","Condition2","Condition2","Condition2"), replicate =
c("Condition1.1","Condition1.2","Condition1.3",..."Condition2.1","Condition2.2","Condition2.3","Condition2.4"))

> mydata <- readData(data = mycounts, factors = myfactors, length = mylength)

> mycountsbio = dat(mydata, factor = NULL, norm=FALSE, type = "countsbio")

> mysaturation = dat(mydata, k = 0, ndepth = 7, type = "saturation")

> QCreport(mydata, samples = NULL, factor = "condition") # This final line produces a quality control report

##################### Repeat with normalized data to see which one works best #################

#######################################################
## No-normalization batch correction
#######################################################

> mycounts<-read.table('gene_counts_CB_vs_PB.tab', header=TRUE, row.names=1)

> mycounts=mycounts[which(rowSums(mycounts) > 0),] # Remove 0S

> mylength<-read.table('gene_length_NOISeq.txt', header=FALSE)

> myfactors = data.frame(condition =
c("Condition1","Condition1","Condition1",..."Condition2","Condition2","Condition2","Condition2"), replicate =
c("Condition1.1","Condition1.2","Condition1.3",..."Condition2.1","Condition2.2","Condition2.3","Condition2.4"))

> mydata2 = readData(mycounts, factors = myfactors, length=mylength)

> mydata2corr2 = ARSyNseq(mydata2, factor = "condition", batch = FALSE, norm = "n",  logtransf = FALSE)
## remove batch effect

> mycountsbio = dat(mydata2corr2, factor = NULL, norm=TRUE, type = "countsbio")

> mysaturation = dat(mydata2corr2, k = 0, ndepth = 7, type = "saturation")

> QCreport(mydata2corr2, samples = NULL, factor = "condition")

#######################################################
## RPKM normalization
#######################################################

> mycounts<-read.table('gene_counts.tab', header=TRUE, row.names=1)
```

```
> mylength<-read.table('gene_length_NOISeq.txt', header=FALSE)

> myrpkm = rpkm(mycounts, long = mylength, lc = 1, k = 0)

> mydata <- readData(data = myrpkm, factors = myfactors)

> mycountsbio = dat(mydata, factor = NULL, norm=TRUE, type = "countsbio")

> mysaturation = dat(mydata, k = 0, ndepth = 7, type = "saturation")

> QCreport(mydata, samples = NULL, factor = "condition")


##########################################################
## RPKM normalization with batch removal
##########################################################

> mycounts<-read.table('gene_counts_BuccalvsTumor.tab', header=TRUE, row.names=1)

> mylength<-read.table('gene_length_NOISeq.txt', header=FALSE)

> myfactors = data.frame(condition =
c("Condition1","Condition1","Condition1",..."Condition2","Condition2","Condition2","Condition2"), replicate =
c("Condition1.1","Condition1.2","Condition1.3",..."Condition2.1","Condition2.2","Condition2.3","Condition2.4"))

> myrpkm = rpkm(mycounts, long = mylength, lc = 1, k = 0)

> mydata2 <- readData(data = myrpkm, factors = myfactors, length=mylength)

> mydata2corr2 = ARSyNseq(mydata2, factor = "condition", batch = FALSE, norm = "n",  logtransf = FALSE)

> mycountsbio = dat(mydata2corr2, factor = NULL, norm=TRUE, type = "countsbio")

> mysaturation = dat(mydata2corr2, k = 0, ndepth = 7, type = "saturation")

> QCreport(mydata2corr2, samples = NULL, factor = "condition")


##########################################################
## TMM normalization with no length correction
##########################################################

> mytmm = tmm(mycounts, long = 1000, lc = 0, k = 0)

> mydata <- readData(data = mytmm, factors = myfactors)

> mycountsbio = dat(mydata, factor = NULL, norm=TRUE, type = "countsbio")

> mysaturation = dat(mydata, k = 0, ndepth = 7, type = "saturation")

> QCreport(mydata, samples = NULL, factor = "condition")
```

```
#######################################################
## TMM normalization with length correction no batch removal
#######################################################

> mycounts<-read.table('gene_counts.tab', header=TRUE, row.names=1)

> mylength<-read.table('gene_length_NOISeq.txt', header=FALSE)

> mytmm = tmm(mycounts, long = mylength, lc = 1, k = 0)

> mydata <- readData(data = mytmm, factors = myfactors)

> mycountsbio = dat(mydata, factor = NULL, norm=TRUE, type = "countsbio")

> mysaturation = dat(mydata, k = 0, ndepth = 7, type = "saturation")

> QCreport(mydata, samples = NULL, factor = "condition")

#######################################################
## TMM normalization with length correction and batch removal
#######################################################

> mycounts<-read.table('gene_counts_BuccalvsTumor.tab', header=TRUE, row.names=1)

> mylength<-read.table('gene_length_NOISeq.txt', header=FALSE)

>  myfactors = data.frame(condition =
c("Condition1","Condition1","Condition1",..."Condition2","Condition2","Condition2","Condition2"), replicate =
c("Condition1.1","Condition1.2","Condition1.3",..."Condition2.1","Condition2.2","Condition2.3","Condition2.4"))

> mytmm = tmm(mycounts, long = mylength, lc = 1, k = 0)

> mydata2 <- readData(data = mytmm, factors = myfactors, length=mylength)

> mydata2corr2 = ARSyNseq(mydata2, factor = "condition", batch = FALSE, norm = "n",  logtransf = FALSE)

> mycountsbio = dat(mydata2corr2, factor = NULL, norm=TRUE, type = "countsbio")

> mysaturation = dat(mydata2corr2, k = 0, ndepth = 7, type = "saturation")

> QCreport(mydata2corr2, samples = NULL, factor = "condition")


#####################################################################################
###################### Differential expression analysis with NOISeqBio in R #####################
#####################################################################################

> library(NOISeq)

> mycounts<-read.table('gene_counts_CB_vs_PB.tab', header=TRUE, row.names=1)

> mycounts=mycounts[which(rowSums(mycounts) > 0),] # Remove 0S
```

```
> mylength<-read.table('gene_length_NOISeq.txt', header=FALSE)

> myfactors = data.frame(condition =
c("Condition1","Condition1","Condition1",..."Condition2","Condition2","Condition2","Condition2"), replicate =
c("Condition1.1","Condition1.2","Condition1.3",..."Condition2.1","Condition2.2","Condition2.3","Condition2.4"))

> mydata2 = readData(mycounts, factors = myfactors, length=mylength)

> mydata2corr2 = ARSyNseq(mydata2, factor = "condition", batch = FALSE, norm = "tmm",  logtransf =
FALSE) ## remove batch effect

> mynoiseq = noiseqbio(mydata2corr2, k = 0.5, norm = "n", factor = "condition", lc = 1, random.seed=12345,
filter=1, cv.cutoff = 50)

> mynoiseq.deg = degenes(mynoiseq, q = 0.95, M = NULL)

> write.table(mynoiseq.deg, file="NOISeqBio_Results_all.txt")

> mynoiseq.deg1 = degenes(mynoiseq, q = 0.95, M = "up") # Upregulated genes 0.95 cutoff is the
recommended by the authors

> write.table(mynoiseq.deg1, file="NOISeqBio_Results_Down.txt")

> mynoiseq.deg2 = degenes(mynoiseq, q = 0.95, M = "down") # Downregulated genes in reference to first
condition

> write.table(mynoiseq.deg2, file="NOISeqBio_Results_Up.txt")

> DE.plot(mynoiseq, q = 0.95, graphic = "expr", log.scale = TRUE) # Plot


########################################################################################
################## GO enrichment analysis with GOSeq in R ##################################
########################################################################################

> library(goseq)

> de.genes <- scan("de_genes.txt", what=character() ) # List of differentially expressed genes

> assayed.genes <- scan("all_genes.txt", what=character() ) # List of all genes assayed in the experiment

> gene.length=scan("gene_lengths.txt", what=numeric() ) # For all genes not only the DE genes

> go.ids= read.table("goIDs.txt",header=FALSE) # Table that assign GO numbers to genes

> gene.vector = as.integer(assayed.genes %in% de.genes)  # Next the gene.vector (0 = not DE 1 = DE) was
created from the gene files

> names(gene.vector) = assayed.genes # Add names to gene.vector

> pwf=nullp(gene.vector,bias.data=gene.length) # Calculate  Probability Weighting Function (PWF)
```

```
> GO.wall=goseq(pwf,gene2cat=go.ids, use_genes_without_cat=TRUE) # Calculate over and under expressed GO categories among DE genes with the wallenius approximation

> FDR_over <-p.adjust(GO.wall$over_represented_pvalue,method="BH") # Calculate FDR values for over_represented FDR
> FDR_under <-p.adjust(GO.wall$under_represented_pvalue,method="BH") # Calculate FDR values for under_represented FDR

> merged.data <- cbind(GO.wall,FDR_over,FDR_under) # Adds FDR values to the table

> write.table(merged.data,"GO_enrichment_Results.txt") # Last column represent the FDR values

> enriched.GO=GO.wall$category[p.adjust(GO.wall$over_represented_pvalue,method="BH")<.05] # Gives list of over represented GO numbers

> write.table(enriched.GO,"GO_over_represented05FDR.txt", row.names=F, col.names=F)

> under.GO=GO.wall$category[p.adjust(GO.wall$under_represented_pvalue,method="BH")<.05]

> write.table(under.GO,"GO_under_represented05FDR.txt", row.names=F, col.names=F)


###########################################################################################
################ Kraken analysis ##########################################################
###########################################################################################

$ ./kraken --db Oral_microbiome/ --threads 35 --fastq-input --gzip-compressed --only-classified-output --output Sample_1.txt --paired Sample_1_R1.fastq.gz Sample_1_R2.fastq.gz

$ ./kraken-mpa-report --db Oral_microbiome/ Sample_1.txt > Sample_1.tab

$ python merge_metaphlan_tables.py Sample_1.tab  .... Sample_NSorted.tab > merged_tables_kraken.txt

$ cut -f2,3 Sample_1.txt > Sample_1.in

$ ./ImportTaxonomy.pl Sample_1.in -o Sample_1.html ###### Generates figure
```
