# Supplementary material for: Increased virulence of the oral microbiome in oral squamous cell carcinoma revealed by metatranscriptome analyses
Source: Int J Oral Sci. 2018 Nov 12;10(4):32. doi: 10.1038/s41368-018-0037-7 (PMC6232154; doi:10.1038/s41368-018-0037-7)
Supplement: Supplementary file 2 — Supplementary Figure 1 [file 41368_2018_37_MOESM2_ESM.pdf]

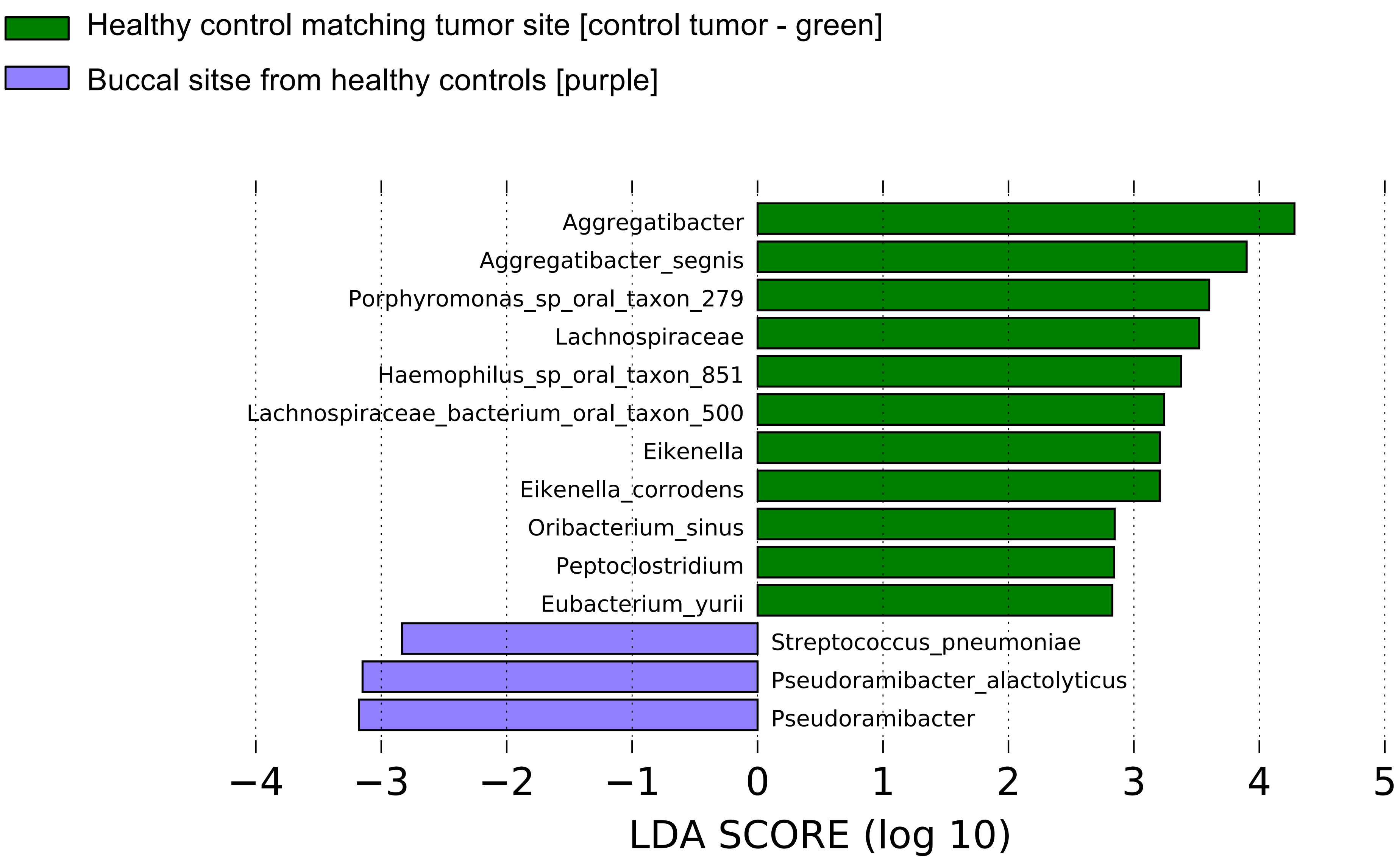

Supplementary Figure 1. Statistical differences in the phylogenetic composition of active communities.
Metatranscriptome hit counts were obtained using Kraken against an oral microbiome database.
Counts were then analyzed using LEfSe to identify significant differences at the species level between the microbial communities compared.
Healthy control matching tumor site [control tumor - green] vs. Buccal site from healthy controls [purple].
