# Supplementary material for: Increased virulence of the oral microbiome in oral squamous cell carcinoma revealed by metatranscriptome analyses
Source: Int J Oral Sci. 2018 Nov 12;10(4):32. doi: 10.1038/s41368-018-0037-7 (PMC6232154; doi:10.1038/s41368-018-0037-7)
Supplement: Supplementary file 3 — Supplementary Figure 2 [file 41368_2018_37_MOESM3_ESM.pdf]

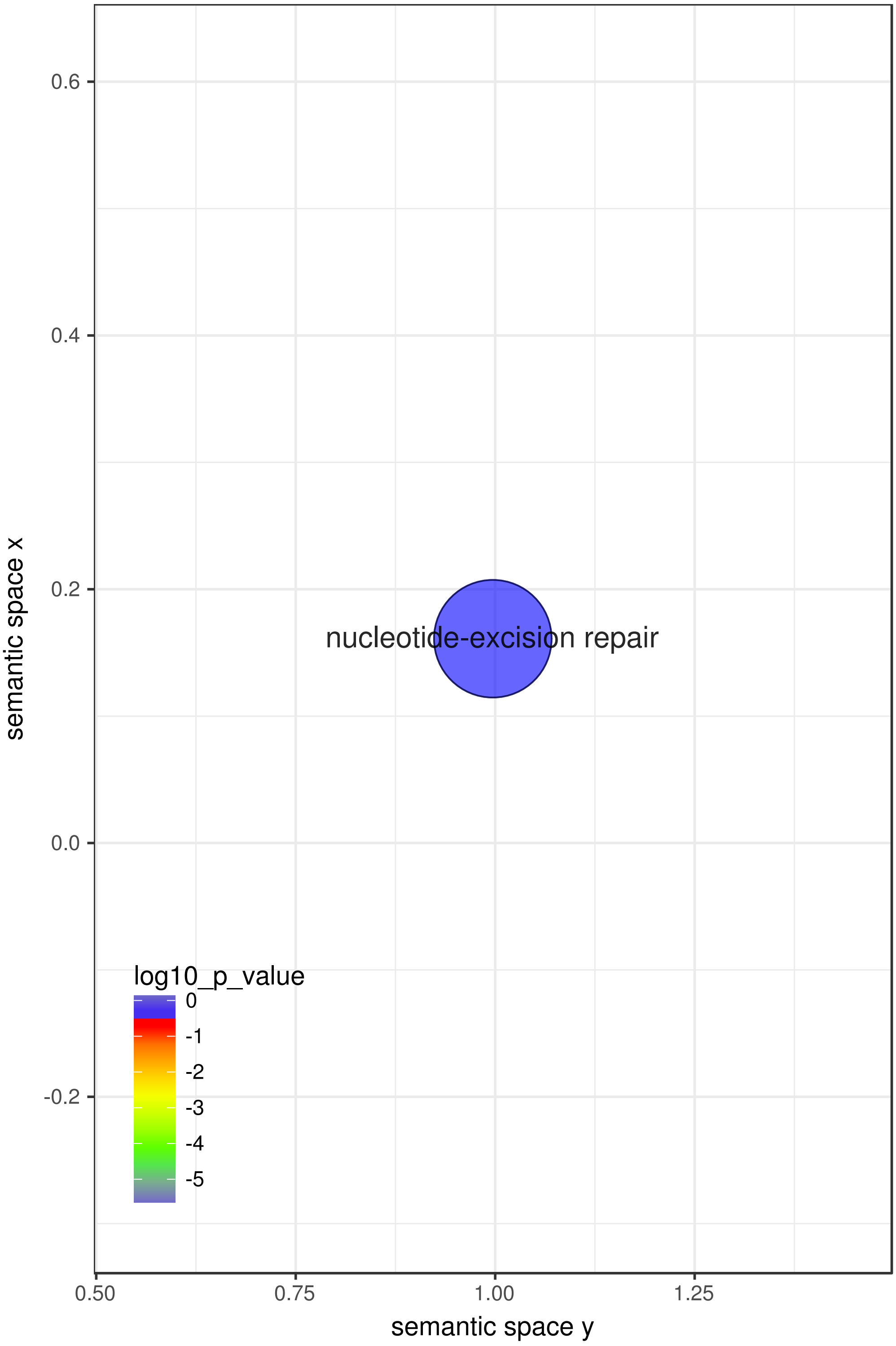

Supplementary Figure 2. GO enrichment analysis for the metatranscriptome profiles of the oral microbiome associated with cancer status; Under-represented Biologic processes.
Enriched terms obtained using GOseq were summarized and visualized as a scatter plot using REVIGO.
Summarized GO terms in PT tumor [tumor site] vs. CT tumor-matched site [control tumor].
Circle size is proportional to the frequency of the GO term, while color indicates the log10 P value (red higher, green lower).
