# Supplementary figures and images for: Increased virulence of the oral microbiome in oral squamous cell carcinoma revealed by metatranscriptome analyses
Source: Int J Oral Sci. 2018 Nov 12;10(4):32. doi: 10.1038/s41368-018-0037-7 (PMC6232154; doi:10.1038/s41368-018-0037-7)

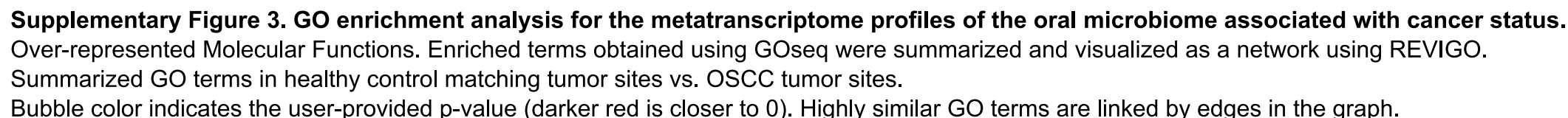

Supplement: Supplementary file 4 — Supplementary Figure 3 [file 41368_2018_37_MOESM4_ESM.pdf]

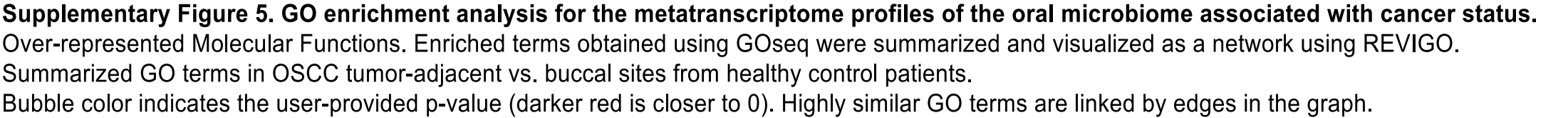

Supplement: Supplementary file 6 — Supplementary Figure 5 [file 41368_2018_37_MOESM6_ESM.pdf]
