# Supplementary material for: Increased virulence of the oral microbiome in oral squamous cell carcinoma revealed by metatranscriptome analyses
Source: Int J Oral Sci. 2018 Nov 12;10(4):32. doi: 10.1038/s41368-018-0037-7 (PMC6232154; doi:10.1038/s41368-018-0037-7)
Supplement: Supplementary file 5 — Supplementary Figure 4 [file 41368_2018_37_MOESM5_ESM.pdf]

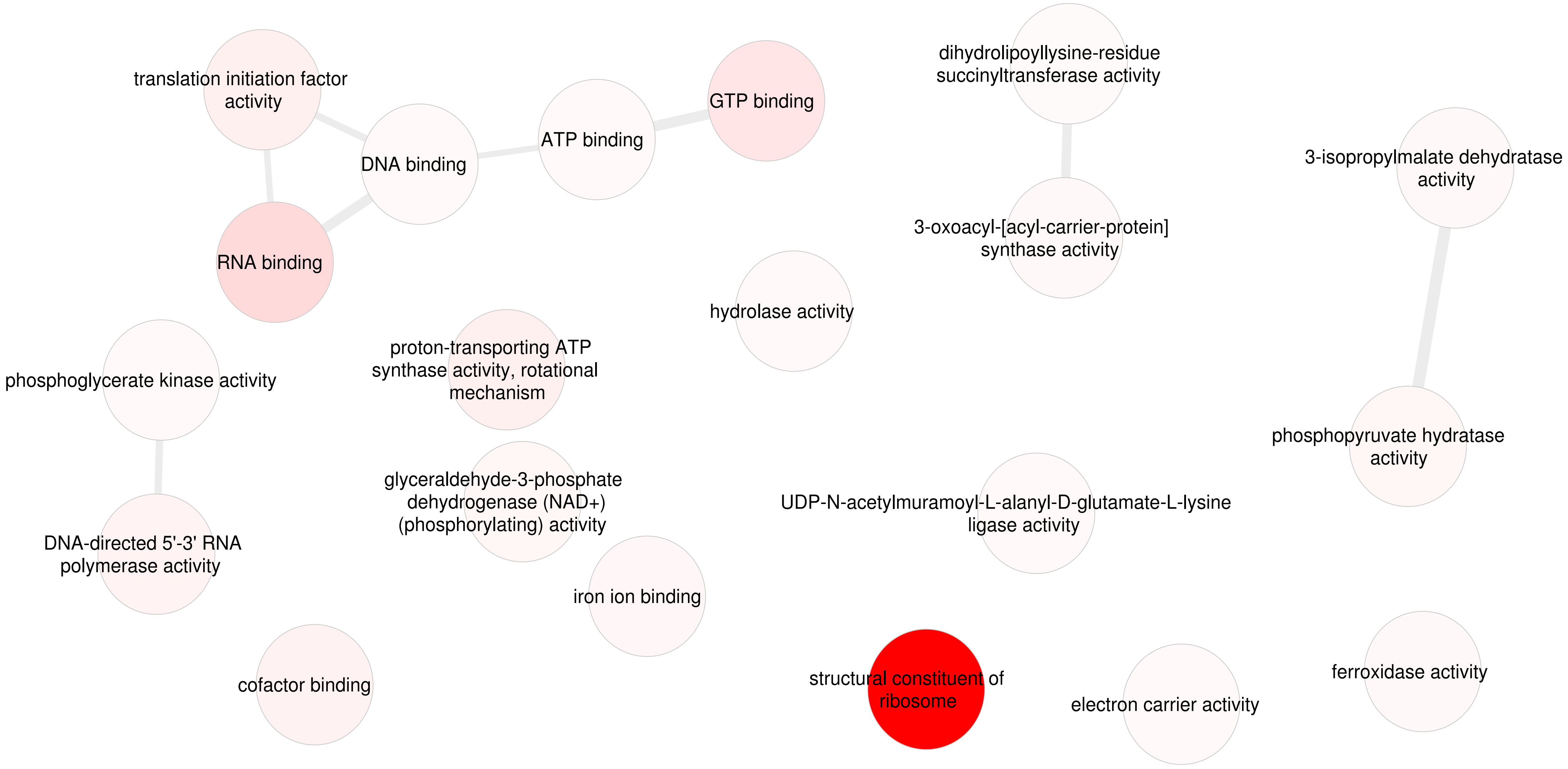

Supplementary Figure 4. GO enrichment analysis for the metatranscriptome profiles of the oral microbiome associated with cancer status. Over-represented Molecular Functions. Enriched terms obtained using GOseq were summarized and visualized as a network using REVIGO. Summarized GO terms OSCC tumor-adjacent vs. OSCC tumor sites.
Bubble color indicates the user-provided p-value (darker red is closer to 0). Highly similar GO terms are linked by edges in the graph.
