# Supplementary material for: Increased virulence of the oral microbiome in oral squamous cell carcinoma revealed by metatranscriptome analyses
Source: Int J Oral Sci. 2018 Nov 12;10(4):32. doi: 10.1038/s41368-018-0037-7 (PMC6232154; doi:10.1038/s41368-018-0037-7)
Supplement: Supplementary file 7 — Supplementary Figure 6 [file 41368_2018_37_MOESM7_ESM.pdf]

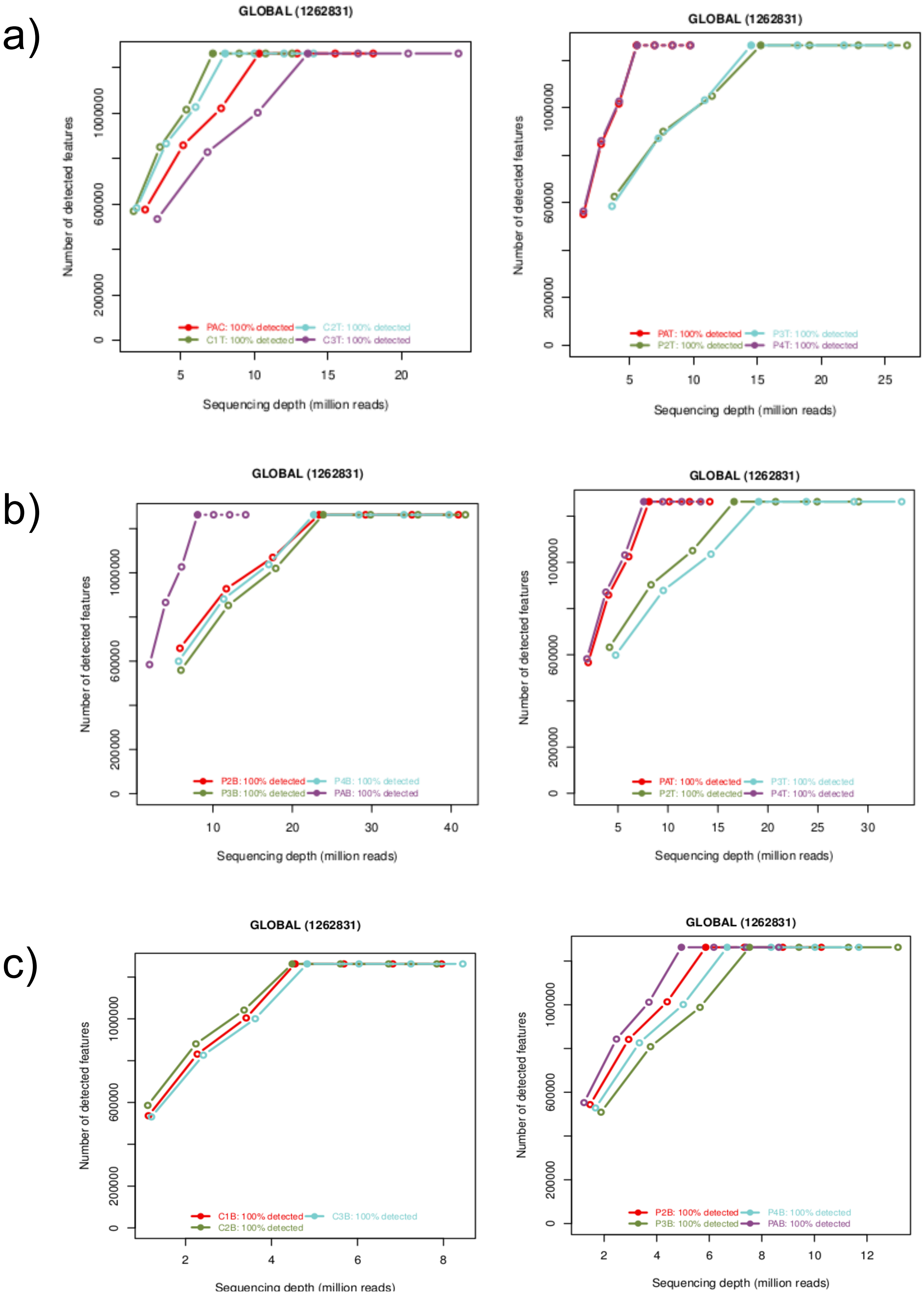

Supplementary Figure 6. Sequencing depth and quantification of expression. QC plots for sequencing depth in the NOISeq package. Graphs show the number of detected genes at the given sequencing depth. a) Control tumor vs. tumor sites. b) Tumor adjacent vs. tumor sites c) Tumor adjacent vs. buccal sites from healthy controls.
