# Supplementary material for: Increased virulence of the oral microbiome in oral squamous cell carcinoma revealed by metatranscriptome analyses
Source: Int J Oral Sci. 2018 Nov 12;10(4):32. doi: 10.1038/s41368-018-0037-7 (PMC6232154; doi:10.1038/s41368-018-0037-7)
Supplement: Supplementary file 8 — Supplementary Table 1 [file 41368_2018_37_MOESM8_ESM.pdf]

Table S1. Sample size computation using RNASeqPower. We first estimated the depth sequencing of the libraries used for analysis. The coefficient of variation used in our calculations was 0.5, the cv cutoff used to filter genes in NOISeqBio analysis. In yellow we indicate the Fold change selected given a sample size of 4 individuals per group.

	**Power=0.8**		
**Fold change**	FDR= 0.1	**FDR = 0.05**	FDR = 0.01
1.5	20.494705	26.018437	38.714888
2	7.012901	8.903018	13.247504
**2.75**	3.292534	4.179937	6.219659
**3.25**	2.425355	3.079036	4.581541
3.5	2.146895	2.725526	4.055526
	**Power=0.9**		
	FDR= 0.1	**FDR = 0.05**	FDR = 0.01
1.5	28.3885	34.831305	49.324033
2	9.714009	11.918615	16.877753
2.75	4.560695	5.59575	7.924049
3.25	3.359511	4.121956	5.837033
3.5	2.973799	3.648706	5.166872
