# Supplementary material for: Increased virulence of the oral microbiome in oral squamous cell carcinoma revealed by metatranscriptome analyses
Source: Int J Oral Sci. 2018 Nov 12;10(4):32. doi: 10.1038/s41368-018-0037-7 (PMC6232154; doi:10.1038/s41368-018-0037-7)
Supplement: Supplementary file 9 — Supplementary Table 2 [file 41368_2018_37_MOESM9_ESM.pdf]

Supplementary Table 2. Cohort characteristics, including disease status and site sampled. Number of total sequences and unique mapped reads to the oral microbiome genome database.

Subject	Disease status	Sample site	Number of sequences	Number of mapped reads
Patient 1	Buccal cancer	Tumor site	48,833,407	36,617,557
		Buccal site no tumor	265,199,761	118,421,522
Patient 2	Floor of mouth cancer	Tumor site	33,933,548	19,080,415
		Buccal site no tumor	14,847,961	8,588,265
Patient 3	Floor of mouth cancer	Tumor site	51,298,649	29,268,699
		Buccal site no tumor	51,065,365	30,700,991
Patient 4	Buccal cancer	Tumor site	6,996,798	3,191,196
		Buccal site no tumor	40,404,375	10,744,928
Control 1	Healthy	Control Tumor site	23,310,479	21,732,254
		Healthy buccal	44,487,026	40,742,641
Control 2	Healthy	Control Tumor site	13,547,239	8,798,109
		Healthy buccal	6,806,791	3,172,602
Control 3	Healthy	Control Tumor site	5,423,938	4,984,258
		Healthy buccal	25,599,665	23,985,334
Control 4	Healthy	Control Tumor site	14,811,504	11,888,368
